# Do remote dialysis services really cost more? An economic analysis of hospital and dialysis modality costs associated with dialysis services in urban, rural and remote settings

**DOI:** 10.1186/s12913-021-06612-z

**Published:** 2021-06-17

**Authors:** Gillian Gorham, Kirsten Howard, Joan Cunningham, Federica Barzi, Paul Lawton, Alan Cass

**Affiliations:** 1 Menzies School of Health Research, Charles Darwin University, PO Box 41096, Casuarina, 0810 Darwin, Australia; 2 Sydney School of Public Health, The University of Sydney, Sydney, Australia

## Abstract

**Background:**

Rates of end-stage kidney disease in Australia are highest in the Northern Territory (NT), with the burden of disease heaviest in remote areas. However, the high cost of delivering dialysis services in remote areas has resulted in centralisation, requiring many people to relocate for treatment. Patients argue that treatment closer to home improves health outcomes and reduces downstream healthcare use. Existing dialysis cost studies have not compared total health care costs associated with treatment in different locations.

**Objective:**

To estimate and compare, from a payer perspective, the observed health service costs (all cause hospital admissions, emergency department presentations and maintenance dialysis) associated with different dialysis models in urban, rural and remote locations.

**Methods:**

Using cost weights attributed to diagnostic codes in the NT Department of Health’s hospital admission data set (2008–2014), we calculated the mean (SD) total annual health service costs by dialysis model for 995 dialysis patients. Generalized linear modeling with bootstrapping tested the marginal cost differences between different explanatory variables to estimate ‘best casemix’/‘worst casemix’ cost scenarios.

**Results:**

The mean annual patient hospital expenditure was highest for urban models at $97 928 (SD $21 261) and $43 440 (SD $5 048) and lowest for remote at $19 584 (SD $4 394). When combined with the observed maintenance dialysis costs, expenditure was the highest for urban models at $148 510 (SD $19 774). The incremental cost increase of dialysing in an urban area, compared with a rural area, for a relocated person from a remote area, was $5 648 more and increased further for those from remote and very remote areas to $10 785 and $15 118 respectively.

**Conclusions:**

This study demonstrates that dialysis treatment in urban areas for relocated people has health and cost implications that maybe greater than the cost of remote service delivery. The study emphasises the importance of considering all health service costs and cost consequences of service delivery models.

**Key points for decision makers:**

Relocation for dialysis treatment has serious health and economic consequences. Relocated people have low dialysis attendance and high hospital costs in urban areas. While remote dialysis service models are more expensive than urban models, the comparative cost differences are significantly reduced when all health service costs are included. The delivery of equitable and accessible dialysis service models requires a holistic approach that incorporates the needs of the patient; hence dialysis cost studies must consider the full range of cost impacts beyond the dialysis treatments alone.

**Supplementary Information:**

The online version contains supplementary material available at 10.1186/s12913-021-06612-z.

## Plain language summary

Most people requiring ongoing treatment for end-stage kidney disease in the Northern Territory (NT) identify as Aboriginal with the majority residing in areas classified as remote or very remote. Unlike other jurisdictions in Australia, haemodialysis in a satellite unit is the most common form of treatment. However, there is a geographic mismatch between demand and service provision, with services centralised in urban areas. Patients and communities have long advocated for services at or closer to home, maintaining that the consequences of relocation and dislocation have far reaching health, psychosocial and economic ramifications. We analysed retrospective hospital data for 995 maintenance dialysis patients, stratified by the model of care they received in urban, rural and remote locations. Using cost weights attributed to diagnosis codes, we costed hospital admissions, emergency department presentations and maintenance dialysis attendances, to provide a mean total health service cost/patient/year for each model of care. We found that urban services were associated with low observed maintenance dialysis and high hospital costs, but the inverse was true for remote and very remote models. Remote models had high maintenance dialysis costs (due to expense of remote service delivery and good dialysis attendance) but low hospital usage and costs. When adjusted for other variables such as age, dialysis vintage and comorbidities, lower total hospital costs were associated with rural and remote service provision. In an environment of escalating demand and constrained budgets, this study underlines the need for policy decisions to consider the full cost consequences of different dialysis service models.

**Supplementary Information:**

The online version contains supplementary material available at 10.1186/s12913-021-06612-z.

## Background

Treatment for end stage kidney disease (ESKD) places an enormous burden on the health system globally and is estimated to cost one billion dollars (AUD) annually in Australia [1]. The treatment for ESKD includes transplantation, haemodialysis (facility-based and home), and peritoneal dialysis, collectively described as renal replacement therapy (RRT). The comparable costs of RRT have been regularly estimated globally, nationally and by jurisdictions, as governments and service providers strive to provide high-cost therapies within increasingly constrained health budgets [2–7].

Self-care therapies of home haemodialysis (HHD) and peritoneal dialysis (PD) are estimated to be the least costly although cost studies vary on the comparative cost differences between HHD and PD. Estimates differ by country, jurisdiction and even locations within States and Territories [8–11]. Self-care therapies are not suited to all individuals and often have a limited treatment life. In Australia most individuals requiring RRT receive haemodialysis in a staffed “satellite” facility, recognised as the most expensive RRT option [12].

Studies have suggested a relationship between the frequency and cost of hospitalisations and specific treatment modalities [13–16]. A few cost studies have also compared the delivery of dialysis treatments across different locations, noting the increased infrastructure and recurrent costs per patient per year associated with remote based services [17–19].

The prevalence of ESKD in Australia is highest in the Northern Territory (NT), with Aboriginal Territorians requiring RRT at more than 10 times the national rate [20]. The burden of kidney disease in the NT is heaviest in remote areas where most Aboriginal Territorians live [21], yet renal and dialysis services are largely centralised in the two urban areas. As a result, most Aboriginal people relocate for treatment, often permanently.

Dialysis service development in rural and remote locations have been limited by concerns regarding the comparatively high establishment and recurrent costs of staffed facilities and the limited capacity of remote primary health services to care for complex conditions, such as ESKD [22–24].

However medical relocation inevitably involves life-changing economic and psychosocial costs, including decreased uptake of RRT [25–27]. While there are strong arguments that improving service accessibility would both improve outcomes and decreases costs [28–30], a rigorous assessment of total health service costs has not been completed to date.

## Objective

The objective of this study was to examine the total health system costs associated with the delivery of dialysis models of care in urban, rural and remote locations from the payer perspective. Health system costs included all cause hospital admissions, emergency department (ED) presentations and maintenance dialysis costs.

Our aim was to estimate and compare the observed mean total costs/patient/year by dialysis model of care and model different casemix scenarios to identify patient characteristics that predicted significant changes in hospital costs.

## Methods

### Overall study design

Australia’s health system is described as a hybrid model. It consists of publicly funded health services based on the premise of universal access to health care, and privately funded services based on user choice [31]. Renal services in the NT are wholly publicly funded as the single private hospital does not provide dialysis treatments. This study took the payer perspective and only included direct health care costs. Patient out of pocket costs, including costs associated with medical relocation were excluded. Costs are reported in 2017 Australian dollars.

We conducted a retrospective analysis of total health service expenditure (dialysis treatments, hospital admissions and ED presentations) for renal patients in the NT between the years 2008–2014. Total health service expenditure was calculated using two approaches:

1. Observed all cause hospital admissions costs, based on the Australian Refined Diagnosis-Related Groups (AR-DRG), a classification system used in Australia for grouping diagnoses/conditions requiring similar hospital services to calculate public hospital funding on an activity basis. Average cost weights are attributed to each episode of care [32]; and.
2. published data of per dialysis treatment costs (inclusive of infrastructure costs) from a recent micro-costing analysis of dialysis program expenditure in the NT [19].

### Setting

The NT, based in northern Australia, is a large land mass with a relatively small, sparsely dispersed population. The majority of the NT is classified as remote or very remote according to the Australian Statistical Geography Standard-*Remoteness* Area (ASGS-RA) classification, which divides Australia into five classes of remoteness based on access to services [33].

Most people live in the two main urban centres of Darwin and Alice Springs, but the majority (70 %) of Aboriginal people, who make up 30 % of the NT population, live in remote/very remote communities [34]. More than 85 % of people receiving RRT in the NT identify as Aboriginal. At the time of this study, most staffed dialysis services were centralised in the urban areas of Darwin and Alice Springs with only limited dialysis services available in remote locations. These small services were usually at capacity with waiting lists. Consequently, 75 % of people receiving care in the urban areas, had moved from very remote areas, often crossing state boundaries to access treatment.

### Dialysis Models of Care

The cohort for this study was established as part of the broader DxMoC [35] project where we conducted a retrospective analysis using deidentified linked clinical and administrative data sets to examine health service utilisation in the NT between 2008 and 2014 (under review). Details of the analysis are available in the electronic supplementary material (ESM). The deidentified data sets were used in this study and all data analysis was performed in accordance with the relevant guidelines and regulations of the relevant ethics committees and in accordance with the Declaration of Helsinki.

We stratified admissions for each patient receiving dialysis in the NT between 2008 and 2014 according to the type and location of dialysis treatment, characterized as a Dialysis Model of Care (DxMoC). We did not include kidney transplantation in the study (see Table 1).

**Table 1:** Dialysis services in the NT characterised as Dialysis Models of Care

*Dialysis Model*	*Description*	*Characteristics*
*DxMoC0* *Incentre dialysis*	Hub service: situated in tertiary centre: acute and maintenance haemodialysis	Majority of patients commence treatment here: used for complex patients and overflow from satellite centres
*DxMoC1* *Urban Satellite Unit*	Large facilities in urban areas: maintenance haemodialysis	All patients stabilised here: default service when rural and remote services at capacity
*DxMoC2* *Rural Satellite Unit*	Smaller facilities: often co-located with regional hospitals: maintenance haemodialysis	Usually for stable patients: generally a waiting list
*DxMoC3* *Remote Satellite Unit*	Small units isolated from hub by distance or geography: maintenance haemodialysis	Generally reserved for clinically well, physically mobile patients: generally a waiting list
*DxMoC4* *Remote CC Satellite Unit*^a^	Aboriginal owned and determined; small remote based units providing permanent and respite dialysis with social supports.	Patient acceptance criteria less restrictive as more support services available: generally a waiting list
*DxMoC5* *SC HD*^b^	Training and support for independent haemodialysis	Clinically stable, deemed capable and safe to deliver own care
*DxMoC6* *SC PD*^c^	Training and support for independent peritoneal dialysis	Clinically stable, deemed capable and safe to deliver own care

^a^*CC *Community controlled, ^b^*SC HD *Self care haemodialysis*, *^c^*SC PD *Self care peritoneal dialysis

### Study cohort

The study population was derived from the NT Department of Health’s Admitted Patient Care (hospital) data set and based on the presence of diagnosis or procedure codes for RRT (International Classification of Diseases Version 10 Australian Modification (ICD 10AM)). The hospital data set contained individual episodes of patient care for the five parent hospitals and satellite dialysis services in the NT. Besides an individual’s demographic details (age, ethnicity, residence), episode data also included the AR-DRG for each admission including maintenance dialysis.

The hospital data set was linked with activity data from: (a) interstate patient travel information (n = 171); and (b) dialysis data from individuals who received care in DxMoC4 and DxMoC5 (n = 189) not captured in the hospital data set. The collection of maintenance dialysis data for DxMoC4 and DxMoC5 was known to be inconsistent, and manual compilation of activity and linkage with the hospital data set was undertaken by an independent linker. Costs were attributed according to the representative AR-DRG.

**Fig. 1:**
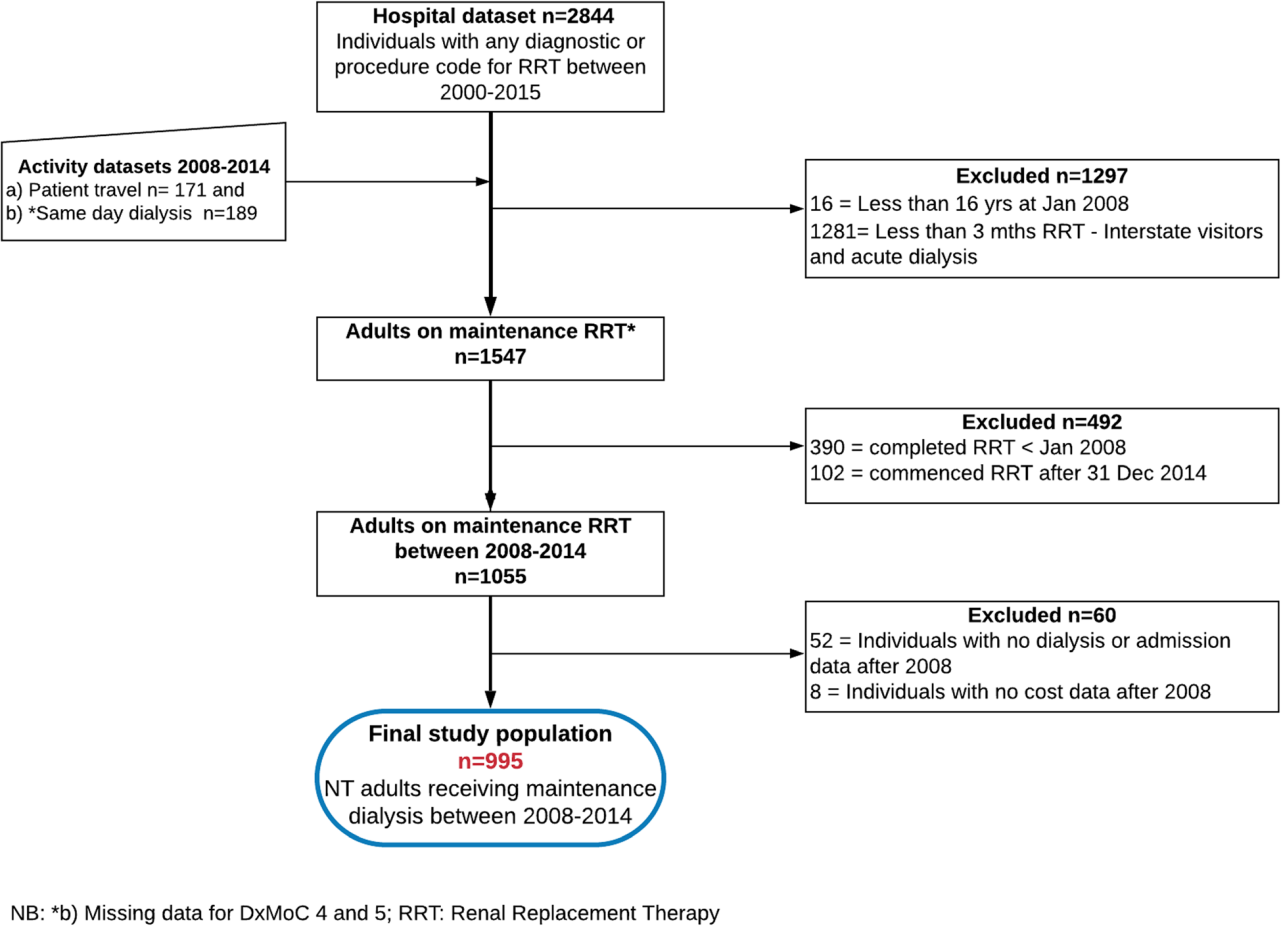
Patient selection flow chart for maintenance dialysis patients 2008–2014

The study population comprised of 995 maintenance dialysis patients, defined as individuals who received dialysis for more than three months continuously (Fig. 1). The analysis was restricted to 2008 to 2014 to ensure sufficient activity was available across all models (new and developing) and to marry with the micro-costing analysis time frame [19]. Eight patients (n = 8) were excluded as they did not have any cost data (Fig. 1). Data was censored at death or withdrawal (based on coding) or loss to follow-up (LTFU) defined as no entry in the data for 12 months or more, without re-entry. Patients who had missing data for 12 months or more (transplanted, moved interstate) but re-appeared in the data set were characterized as intermittent LTFU (iLTFU) for that period.

### Data management

The data was checked and cleaned to ensure consistency of demographic variables (such as gender and ethnicity) and duplicate attendances removed. Same day attendance at both a dialysis facility and the ED, which did not result in an overnight admission (n = 3716) were retained as these records represent additional quantifiable resource allocations.

Variables for the presence of select comorbidities prevalent in ESKD and considered to have a significant impact on health outcomes were created (diabetes, cardiac disease, vascular disease, hypertension, cerebrovascular disease and obesity) based on the presence of relevant ICD 10AM codes. These were carried forward to subsequent episodes once present. Other variables that were considered relevant included region (Top End and Central Australia), time on dialysis, age at admission, Aboriginal status and remoteness of residence prior to commencement of RRT start.

In the NT, there is significant movement between DxMoC with variability across models in the number and proportion of patients that spend a full year in any DxMoC (Table 2). When mapped, DxMoC0 had the lowest proportion of patients (3 %) with a full year, with a median *time in model* of 0.33 year (IQR: 0.13–0.67). This was not unexpected given the model is primarily reserved for new patients and complex care. Similarly, DxMoC4, primarily a respite model, only had 9 % of patients with a full year and a median *time in model* of 0.76 year (IQR:0.36–0.94).

Due to this mobility, the attribution of hospital costs to a specific DxMoC required careful analysis and testing of approaches. The dominant dialysis model of care for each patient was based on rolling attendance over three weeks. This was designed to reduce the “noise” of frequent movement between models while still enabling the respite model (DxMoC4) of 2 to 3 weeks to be captured in the data.

**Table 2:** Patients experiencing full year in DxMoC 2008–2014 with mean (95 % CI) and median (IQR) time

DxMoC and patients (n)	Full year n (%)	Mean time (95 % CI)	Median time (IQR)
DxMoC0 (*n* = 692)	18 (3 %)	0.43 (0.42–0.43)	0.33 (0.13–0.67)
DxMoC1 (*n* = 865)	473 (55 %)	0.88 (0.88–0.88)	1 (0.86-1)
DxMoC2 (*n* = 199)	102 (51 %)	0.90 (0.90–0.90)	1 (0.94-1)
DxMoC3 (*n* = 41)	24 (59 %)	0.89 (0.88–0.89)	1 (0.88-1)
DxMoC4 (*n* = 131)	12 (9 %)	0.66 (0.65–0.66)	0.76 (0.36–0.94)
DxMoC5 (*n* = 128)	49 (38 %)	0.87 (0.86–0.87)	1 (0.81-1)
DxMoC6 (*n* = 158)	86 (54 %)	0.85 (0.85–0.86)	1 (0.81-1)

### Person years

Time (weeks) spent in a DxMoC per year was calculated as the exposure time specific to a model of care and was used in the calculation of person years. Exposure time was censored at death or permanent loss to follow-up. Person years was calculated for each DxMoC and for each year of the study. The full list of variables (definitions and calculations) can be found in the ESM.

### Cost data management

Costs were attributed to each admission according to the AR-DRG. Seven (7) admissions were uncoded, with neither AR-DRG or ICD 10AM codes present. These were allocated a generic AR-DRG based on the length of hospital admission.

Dummy cost variables were created to identify and differentiate the dialysis costs between the different DxMoC not represented in the hospital data set. For instance, while maintenance dialysis as an outpatient is captured for each treatment as a same day admission, the cost differential between different models and regions are not adequately recognised in the case mix funding model. The cost for each same day dialysis at each DxMoC was replaced with the relevant and representative cost from the micro costing study [19]. Incentre haemodialysis (DxMoC0) is accepted as being more expensive than satellite dialysis (DxMoC1) [36, 37]. As DxMoC0 was not included in the micro-costing study, we allocated the DxMoC1 cost value from the study inflated by 17 %, based on the incremental cost differences between DxMoC1 and DxMoC0 from the available research [1, 8, 36–40].

Costs for self-care training for both DxMoC5 (Self-care HD) and DxMoC6 (Self-care PD) were only applied to incident patients (those starting RRT after January 2008), and only in the first year of allocation to the model. The training cost for DxMoC5 was based on findings from the micro-cost study which included a lengthy training period of up to six months. Yearly DxMoC6 costs were applied as a proportion of the time spent in the model. The replacement costs for AR-DRG code L61Z (same day haemodialysis) and one-off costs applied for DxMoC6 (peritoneal dialysis) based on the representative values from the micro costing study [19] are in Table 3.

**Table 3:** Replacement unit costs for same day haemodialysis and peritoneal dialysis activity, $2017

Model of care and region*	**Replacement unit cost for AR-DRG L61Z** [13]	**Applied**
DxMoC0 - TE^1a^	$682.41	All patients in model
DxMoC0 - CA^1a^	$662.59	All patients in model
DxMoC1 - TE	$583.26	All patients in model
DxMoC1 - CA	$566.32	All patients in model
DxMoC2 - TE	$519.29	All patients in model
DxMoC2 - CA	$565.70	All patients in model
DxMoC3 - TE	$798.03	All patients in model
DxMoC4 - CA	$770.33	All patients in model
DxMoC5 - TE (training)	$579.17	Incident patients to DxMoC5 - applied to first 180 days in model
DxMoC5 - TE post training	$275.17	Prevalent patients and after first 180 days in model for incident patients
DxMoC5 - CA (training)	$850.85	Incident patients to DxMoC5 - applied to first 180 days in model
DxMoC5 - CA post training	$435.90	Prevalent patients and after first 180 days in model for incident patients
DxMoC6 - TE (training)	$33,260.00	Incident patients to DxMoC6 - one off amount in first year DxMoC6 appears
DxMoC6 - TE post training	$58,489.18	Prevalent patients with annual cost proportioned to time in DxMoC6 (exclusive of 6 weeks training time)
DxMoC6 - CA (training)	$17,854.00	Incident patients to DxMoC6 - one off amount in first year DxMoC6 appears
DxMoC6 - CA post training	$61,427.59	Prevalent patients with annual cost proportioned to time in DxMoC6 (exclusive of 6 weeks training time)

*Region: TE: Top End, *CA:* Central Australia; ^1a^ Based on DxMoC1 costs inflated by 17% - using average incremental cost difference between DxMoC1 (urban services) and DxMoC0 (incentre facility) from seven studies [28–31]

### Statistical analysis

All analyses were conducted using Stata15.1© (StataCorp., College Station; Texas USA). Descriptive statistics included the total mean and standard deviation for all health service costs (overnight admissions, ED presentations and maintenance dialysis costs) and health service costs per person year.

We considered and tested a range of models for goodness of fit including a two part generalized linear model (GLM) [41]. We explored the completeness of costs for each individual and by DxMoC to determine the level of ‘missingness’ and if cost imputation was required [42]. We used a standard GLM (as the inclusion of zero costs was important in this context) to test marginal effects and a modified Park Test to determine the link family [43]. The GLM with log and gamma family (and a cluster option to account for multiple observations) was then used to test the marginal cost differences between different explanatory variables to create models to predict ‘best casemix’/‘worst casemix’ cost scenarios [44]. Bootstrapping of 2000 repetitions was used to test the robustness of cost outputs and to account for the unevenness of utilisation between DxMoC.

## Results

### Study population

Of the nine hundred and ninety-five individuals who met the inclusion criteria, there were more females than males, with Aboriginal people making up the majority of the study cohort (90 %). There was homogeneity across age at study entry and median study time with little variance by gender and Aboriginal status.

Overall, 75 % of the study population had a residential address outside of the urban areas of Darwin and Alice Springs (greater than 90 min drive) prior to the start of RRT. The proportion of people with a recorded comorbidity of diabetes and cardiac disease was more common in Aboriginal people than non-Aboriginal people (Table 4).

**Table 4:** Patient characteristics by first episode in data set by gender and Aboriginal status

**Characteristics of cost study cohort **	***n=995***	**Aboriginal n = 892 (90%)**		**Non-Aboriginal n = 103 (10%)**	
		Female	Male	Female	Male
Gender (female) n (%)	566 (57%)	526 (59% )	366 (41%)	40 (39%)	63 (61%)
Mean age at study entry (SD)	51 (11.7)	51 (11.4)	50 (11.0)	50 (16.0)	56 (14.7)
Median study time (IQR)	3.2 (1.5-5.7)	3.3 (1.4-5.8)	3.2 (1.5-5.6)	3.2 (1.6-5.7)	3.1 (1.3-4.9)
**Relocation**					
Relocated n (%)	743 (75%)	429 (82%)	304 (83%)	4 (10%)	6 (10%)
Residence pre RRT (very remote)	703 (71%)	407 (77%)	288 (78%)	3 (7%)	5 (8%)
**Comorbidities at study entry **					
Diabetes	767 (77%)	439 (84%)	287 (79%)	20 (49%)	21 (33%)
Cardiac Disease	480 (48%)	256 (49%)	188 (51%)	15 (37%)	21 (33%)
Vascular Disease	333 (33%)	183 (35%)	118 (32%)	9 ( 22%)	23 (37%)
Obesity	116 (12%)	58 (11%)	50 (14%)	4 (10%)	4 (6%)
Hypertension	910 (91%)	487 (93%)	339 (93%)	34 (85%)	50 (79%)
Cerebrovascular Disease	49 (5%)	24 (5%)	25 (7%)	0 (0%)	5 ( 8%)

### Health service utilisation

Health service costs were separated by overnight hospital admissions, ED presentations not resulting in an overnight admission and maintenance dialysis (same day haemodialysis and annual (pro rata) peritoneal dialysis expenditure). Overnight admission and ED costs are referred to collectively as ‘hospital service’ costs.

Expenditure was examined for each patient, each year and by DxMoC. While our analysis used methodology that allowed prediction of costs, our primary objective was to determine the relative overall observed costs for each DxMoC. Approximately 80 % of patients incurred at least one overnight admission each year and 35 % presented to the ED at least once in a year. Approximately 15 % of patients each year had neither a hospital admission nor ED presentation and therefore had zero health service costs other than maintenance dialysis costs. Health service activity for patients in each year is presented in Table 5.

**Table 5:** Health service activity for patients active in each year excluding same day dialysis

**Health service use**	***2008 (n=474)***	***2009 (n=483)***	***2010 (n=511)***	***2011 (n=548)***	***2012 (n=587)***	***2013 (n=621)***	***2014 (n=647)***
Admitted patients n (%)	378 (80%)	380 (79%)	411 (80%)	440 (80%)	467 (80%)	502 (81%)	528 (82%)
Overnight admission episodes = *n*	1325	1485	1724	1725	1756	2009	2376
Patients presenting to ED^a^ n (%)	164 (35%)	178 (37%)	169 (33%)	182 (33%)	196 (33%)	226 (36%)	247 (38%)
ED presentation episodes = *n*	377	449	487	492	515	689	727
Zero hospital and ED presentations *n* (%)	72 (15%)	71 (15%)	74 (14%)	84 (15%)	81 (14%)	88 (14%)	79 (12%)

^a^Emergency Department

The completeness of activity for each patient was examined to determine reasons for partial activity. Approximately 70 % of patients each year had a full year of activity. Partial year costs were due to a variety of reasons including incidence, death and loss to follow-up (LTFU). Patients who did not have a full year of activity in 2014 but were expected to (that is, were not transplanted, deceased or on DxMoC6) were characterized as LTFU. This is likely to have inflated the counts for LTFU in 2014 as it is not possible to know whether the individuals reappeared in the data set in 2015 (Table 6).

**Table 6:** Number and proportion of patients with complete and partial year costs by year

**Year of study and patient numbers **	**2008 (n = 474)**	**2009 (n = 483)**	**2010 (n = 511)**	**2011 (n = 548)**	**2012 (n = 587)**	**2013 (n = 621)**	**2014 (n = 647)**
Patients with complete year costs n (%)	327 (69%)	363 (75%)	388 (76%)	404 (74%)	431 (73%)	448 (72%)	439 (68%)
Part year costs due to							
*Incident*	80 (17%)	62 (13%)	64 (12%)	79 (14%)	89 (15%)	94 (15%)	92 (14%)
*Death*	48 (10%)	34 (7%)	39 (8%)	37 (7%)	43 (7%)	42 (7%)	58 (9%)
*Withdrew*^d^	5 (1%)	10 (2%)	5 (1%)	7 (1%)	2 (0%)	10 (%1)	9 (1%)
*LTFU*^b^	1 (0%)	1 (0%)	0 (0%)	6 (1%)	5 (1%)	14 (3%)	43 (7%)
*iLTFU*^a^* including Tx*^c^	13 (3%)	13 (3%)	15 (3%)	15 (3%)	17 (4%)	13 (2%)	6 (1%)

^a^*iLTFU *Intermittent Loss to follow up, ^b^*LTFU Loss to follow up, *^c^*Tx Transplantation, *^d^*Withdrew: palliated*

We determined that partial year costs were not due to missing data (missing at random (MAR)) [42] and the analysis by person years would account for incomplete years when determining the mean costs. We did not impute missing costs.

### Health Service Costs

Hospital service costs were positively skewed. The crude mean annual hospital service expenditures stratified by DxMoC, are presented in Table 7. All costs are in $2017 Australian dollars.

**Table 7:** Average annual health service expenditure by DxMoC and by patient years (2008-2014) $AUS2017

**DxMoC costs**	**Incentre MoC0 **	**Urban DxMoC1 **	**Rural MoC2 **	**Remote MoC3 **	**RemoteCC MoC4 **	**SC HD MoC5 **	**SC PD MoC6**
**Mean annual person years (SD)**	26.9 (3.5)	293.2 (33.9)	65.5 (9.9)	15.2 (3.3)	20.2 (9.9)	33.5 (7.7)	42.6 (8.2)
**Annual overnight costs (SD) **	$2 384 554 ($292 382)	$10 796 590 ($2 338 105)	$2 057 456 ($536 526)	$298 030 ($110 777)	$400 455 ($198 295)	$783 561 ($262 117)	$1 195 783 ($267 061)
**Annual ED cost (SD)**	$197 395 ($69 154)	$2 080 762 ($576 588)	$161 867 ($86 244)	$5 456 ($6 402)	$104 606 ($61 532)	$37 391 ($16 678)	$67 633 ($23 333)
**Annual hospital (overnight and ED) costs (SD)**	$2 581 949 ($339 333)	$12 887 350 ($2 897 673)	$2 219 323 ($607 166)	$303 486 ($115 358)	$505 060 ($252 482)	$820 952 ($267 606)	$1 263 416 ($276 490)
**Annual dialysis costs (SD)**	$1 376 173 ($267 504)	$20 648 550 ($2 412 165)	$4 787 821 ($743 545)	$1 702 762 ($322 872)	$2 140 761 ($1 048 406)	$1 496 354 ($314 784)	$2 986 276 ($554 379)
**Total annual health service costs (SD)**	$3 958 122 ($391 353)	$33 525 910 ($5 185 195)	$7 007 144 ($1 244 222)	$2 006 248 ($399 797)	$2 645 821 ($1 296 984)	$2 317 ($531 984)	$4 249 692 ($673 865)
^a^**Mean hospital costs/patient/yr (SD)**	*$97 928 ($21 261)*	*$43 440 ($5 048)*	*$33 630 ($7 213)*	*$19 584 ($4 394)*	*$24 914 ($4 537)*	*$24 432 ($6 618)*	*$30 699 ($8 898)*
^a^**Mean dialysis costs/patient/yr (SD)**	*$50 582 ($4 434)*	*$70 418 ($1 169)*	*$73 118 ($1 388)*	*$112 696 ($6 322)*	*$105 932 ($3 556)*	*$44 959 ($4 067)*	*$70 391 ($4 272)*
^a^**Mean health service costs/patient/yr (SD)**	*$148 510 ($19 774)*	*$113 858 ($4 891)*	*$106 748 ($6 364)*	*$132 280 ($3 159)*	*$130 846 ($5 280)*	*$69 391 ($8 169)*	*$101 090 ($9 421)*

^a^Annual costs divided by person years

**Fig. 2:**
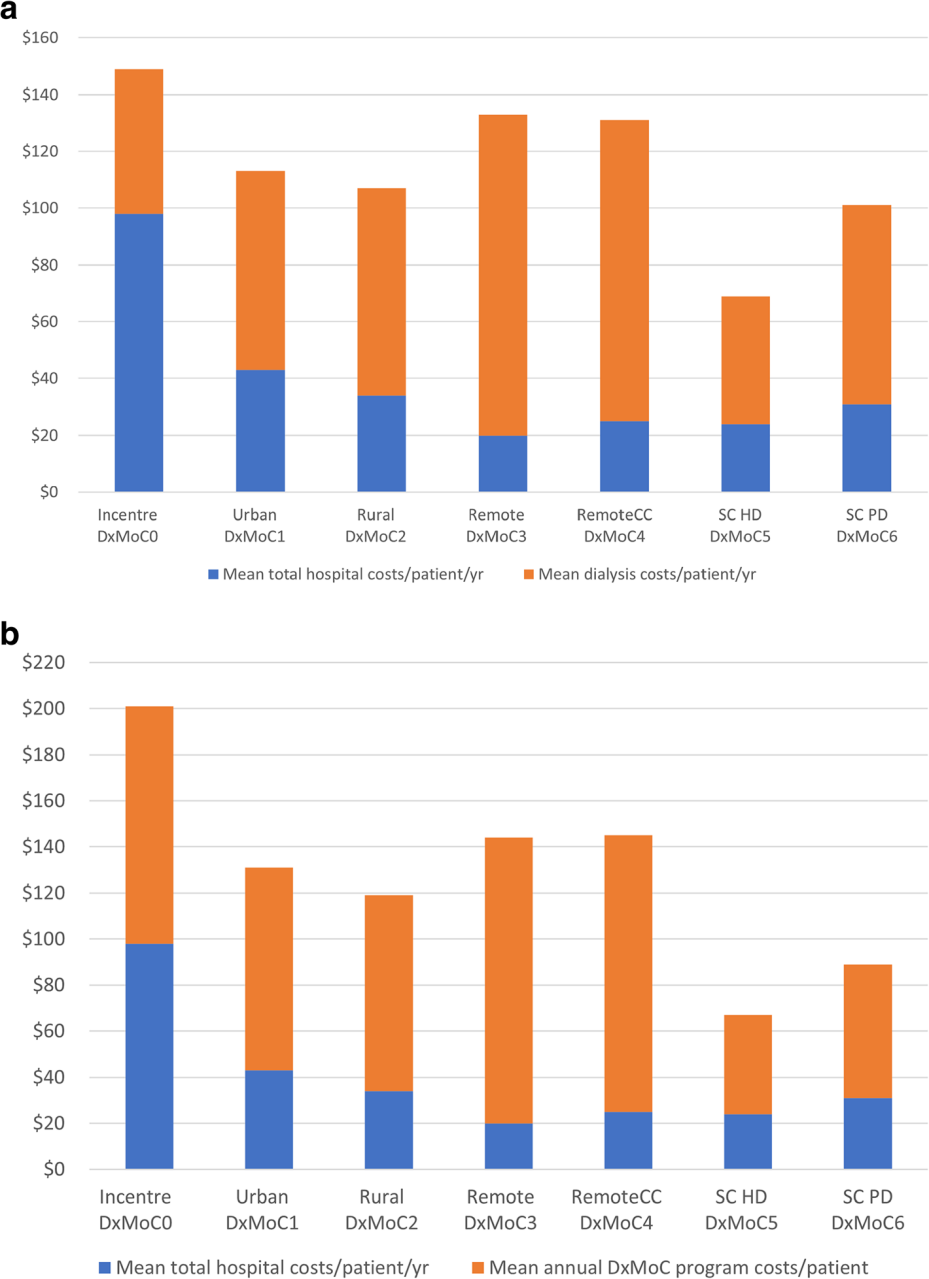
**a** Observed mean annual hospital and maintenance dialysis costs/patient/year. **b** Observed mean annual hospital costs/patient/year with annual patient maintenance dialysis costs based on full annual attendance in a DxMoC from micro-costing analysis [19].

Mean annual hospital patient costs (calculated by dividing total hospital service costs by person years) for each DxMoC were highest for DxMoC0, with a mean cost of $97 928 (SD: $21 261) and lowest for DxMoC3 with a mean cost of $19 584 (SD: $4 394). When combined with the observed maintenance dialysis costs, expenditure was the highest for DxMoC0 at $148 514 (SD $19 774) followed by DxMoC3/4 at $132 280 and $130 846, respectively (Fig. 2 a). Where the annual dialysis patient costs from the micro-costing study [19] were used instead of the observed maintenance dialysis costs, total costs were generally consistent (within 10–15 %) for all DxMoC except DxMoC0 and DxMoC1 (Fig. 2 b). The observed annual dialysis expenditure per patient was lower by 50 and 20 % for DxMoC0 and DxMoC1 respectively (Fig. 2 a), compared to the micro-costing analysis (Fig. 2 b), due to the lower observed attendances in these DxMoC.

### Case mix factors in health service costs

Using GLM regression we predicted low cost and high cost casemix scenarios based on the marginal and incremental costs attributed to different exposure variables. As DxMoC1 had the largest patient population and activity, this model was used as the comparative reference for all other DxMoC. Region, dialysis vintage, comorbidities of diabetes, cardiac disease and vascular disease and DxMoC were significant in the modelling (P < 0.001). Results are presented for Aboriginal people as they made up the majority of the cohort (90 %), and all attendances at DxMoC2-4.

We predicted the costliest casemix would include male gender, relocated from a very remote border community for dialysis treatment, dialysis vintage of less than 12 months, age between 30 and 39 years, with a comorbid condition of vascular disease and receiving care within the Central Australian (CA) region at DxMoC0 or DxMoC1. This casemix incurred between $88 635 and $91 935 in hospitalisation costs each year.

In contrast, the low cost casemix scenario included demographic factors such as an older (between 50 and 59 years of age) female, from the urban area, on dialysis for 4–5 years, dialysing in the Top End (TE) region at DxMoC5 (self-care HD). Hospital costs were predicted to be significantly lower ($22 434), and this remained the case even if a major comorbidity such as vascular disease, was included ($33 158).

We used 50–59 years as the age reference group as this was close to the mean age of the study cohort (Table 4). For each model, we noted costs decreased with each older age group and with each dialysis vintage grouping (Table 8).

High cost casemix scenarios included remote residence pre RRT, being new to dialysis (less than 12 months), relatively young (30–39 years), with a comorbidity such as vascular disease and dialysing in the urban area (DxMoC0/1) in CA. Other than presence of a select comorbidity, this combination of factors is common in Northern Territory dialysis patients. This casemix predicted hospital costs between $100 079 and $104 777 annually.

The lowest cost casemix could be attributed to a combination of characteristics that included an older (50–59 years) female, with no comorbidities who had been on dialysis for 4–5 years and either originally from the urban area and undertaking DxMoC5 OR originally from the remote area and dialysing in DxMoC4.

At the very least, the incremental cost for an Aboriginal person dialysing in a rural (DxMoC2) area was $5 694 lower, compared with a relocated Aboriginal person (with all other things being equal) dialysing in the urban area (DxMoC1). The incremental cost reduction compared to urban dialysis was dependent on remoteness, with DxMoC3 having, on average, $10 818 lower costs than DxMoC1, and DxMoC4 having $15 125 lower costs than DxMoC1 (Table 8).

**Table 8:** Mean annual costs for Aboriginal dialysis patients by included variables $AUS2017

**Multivariate GLM regression for Aboriginal people with included variables **	**Marginal differences (95% CI)**	***P*** value
Constant	$25 797 ($24 793 to $26 801)	<0.001
Annual time in model	$39 189 ($34 558 to $43 819)	<0.001
Gender		
Male	$2 594 ($477 to $4 712)	0.016
Region		
Top End (vs Central Australia)	-$5 578 (-$7 943 to -$3 214)	<0.001
Residence Pre RRT		
Urban	Reference	
Remote	$5 451 ($754 to $10 076)	0.023
Very remote	$4 777 ($1 312 to $8 243)	0.007
Remote border communities	$16 594 ($2 605 to $30 584)	0.020
Admission age		
<30yrs	$9 405 ($2 950 to $15 860)	0.004
30-39yrs	$13 381 ($8 773 to $17 989)	<0.001
40-49yrs	$3 977 ($1 506 to $6 844)	0.002
50-59yrs	Reference	
60-69yrs	$1 442 (-$1 204 to $4 090)	0.285
>70yrs	$1 213 (-$4 129 to $6 556)	0.656
Time on dialysis		
<12mths	Reference	
1-2 yrs	-$9 707 (-$14 288 to -$5 127)	<0.001
2-3yrs	-$11 548 (-$16 750 to -$6 345)	<0.001
3-4yrs	-$10 524 (-$16 291 to -$4 757)	<0.001
4-5yrs	-$14 628 (-$20 021 to -$9 234)	<0.001
>5yrs	-$13 951 (-$18 819 to -$9 083)	<0.001
Comorbid conditions		
Diabetes (vs no diabetes)	$4 614 ($1 380 to $7 847)	0.005
Cardiac (vs no cardiac)	$8 632 ($6 371 to $10 892)	<0.001
Vascular (vs no vascular)	$11 930 ($9 524 to $14 336)	<0.001
DxMoC		
Incentre DxMoC0	$4 702 ($742 to $8 662)	0.020
Urban DxMoC1	Reference	
Rural DxMoC2	-$5 694 (-$8 493 to -$2 894)	<0.001
Remote DxMoC3	-$10 818 (-$15 468 to -$6 167)	<0.001
Remote CC DxMoC4	-$15 125 (-$18 561 to -$11 689)	<0.001
SC HD DxMoC5	-$8 854 (-$13 412 to -$4 296)	<0.001
SC PD DxMoC6	-$2 093 (-$6 468 to $2 280)	0.348

### Discharge diagnosis for DxMoC

We examined the type and frequency of admissions (based on AR-DRG discharge codes) to better understand what other factors impact on DxMoC hospital costs. There was variation in the most frequent discharge diagnosis between models. Kidney related conditions without complications was the most frequent hospitalisation AR-DRG for DxMoC0,1 and 4, while AR-DRGs for DxMoC2 and 3 were respiratory related conditions with severe to moderate (but not catastrophic) complications. These conditions had cost weights twice as high as those for kidney related conditions (Table 9). However, discharge codes associated with DxMoC5 and 6 tended to be more acute (infections and obstructions) with severe or catastrophic complications and a cost weight three times that of the kidney related conditions. When the costs for the top three admission codes for each DxMoC were aggregated, the average per episode cost was considerably higher in DxMoC5 and 6 compared to all other models (Table 9).

**Table 9:** Top three discharge diagnoses for each DxMoC (n (%)), cost weights per episode aggregated and averaged

**AR-DRG**	**AR-DRG Description**	**Cost weight**	**DxMoC0**	**DxMoC1**	**DxMoC2**	**DxMoC3**	**DxMoC4**	**DxMoC5**	**DxMoC6**
L65B	Kidney and Urinary Tract Signs and Symptoms W/O Catastrophic or Severe CC	$2,758	277 (15%)	2036 (18%)	90 (5%)	6 (4%)	47 (13%)	23 (5%)	
L65A	Kidney and Urinary Tract Signs and Symptoms W Catastrophic or Severe CC	$7,246	222 (12%)	979 (9%)					
L67A	Urinary Stones and Obstruction W Catastrophic or Severe CC	$9,985							104 (11%)
K62B	Miscellaneous Metabolic Disorders W/O Catastrophic or Severe CC	$4,691		355 (3%)					
E62B	Respiratory Infections/Inflammations W Severe or Moderate CC	$6,917			99 (6%)	5 (3%)			
Z64A	Other Factors Influencing Health Status	$4,875			97 (6%)				
L60C	Kidney Failure W/O Catastrophic or Severe CC	$3,970	95 (5%)						
T64B	Other Infectious and Parasitic Diseases W Severe or Moderate CC	$9,167						60 (12%)	
L67B	Other Kidney and Urinary Tract Disorders W/O Catastrophic or Severe CC	$4,563							57 (6%)
L09A	Other Procedures for Kidney and Urinary Tract Disorders W Cat CC	$29,964							51 (5%)
F74Z	Chest Pain	$5,326					26 (7%)		
G66Z	Abdominal Pain or Mesenteric Adenitis	$2,378					16 (5%)		
F75B	Other Circulatory Disorders W Severe or Moderate CC	$6,168						16 (3%)	
E65B	Chronic Obstructive Airways Disease W/O Catastrophic CC	$5,886				10 (6%)			
**Average cost per admission -Top 3 admissions aggregated**			**$4,629**	**$4,143**	**$4,916**	**$5,238**	**$3,440**	**$7,193**	**$13,334**

## Discussion

Maintenance dialysis is the most common form of RRT for Aboriginal people in the NT and throughout Australia. There is an abundance of literature on the cost of delivering different modalities; assessments have also been made on the broader health service cost consequences of each modality [45–47]. There is limited literature on the cost implications of dialysis delivery in different locations that also considers broader health service use.

Our findings support previous work that found annual maintenance dialysis costs were higher for rural and remote based DxMoC (DxMOC2-4) compared to urban based (DxMoC0/1) or self-care modalities (DxMoC5/6) [19]. However, this study also demonstrated that the costs associated with overnight admissions and ED presentations were much lower in these models. Based on observed individual attendances, the annual maintenance dialysis costs for DxMoC0 were lower than expected. Non-attendance for dialysis will result in lower maintenance dialysis costs, but as seen in this study, the downstream consequences include frequent admissions and higher overall health service costs. Frequent admissions and poor health also have an impact on quality of life and this in turn has cost consequences [48].

While the findings support our previous analysis (under review) that higher hospital presentations (and therefore costs) observed in DxMoC0 and DxMoC1 were less related to the characteristics of patients attending these models (such as age or comorbidities) than to dialysis attendance patterns, we acknowledge there are other reasons for hospitalisations. Most overnight episodes of care were for relatively uncomplicated, kidney related, short stays. However, the self-care therapies (DxMoC5/6) had lower observed admissions, but the discharge diagnoses and costs indicated these admissions were both more severe and complex, consistent with findings reported elsewhere [49, 50].

The casemix analysis examined patient characteristics that predict significant changes in hospital costs and found individual comorbidities and remoteness of residence pre RRT start contributed significant marginal costs. However, all else being equal, model of care was a significant contributing factor, with incremental costs for all DxMoC lower when compared to DxMoC0/1. The incremental cost difference was the largest for remote services (DxMoC3 and4) and the smallest (and non-significant) for DxMoC6.

Our study was from the funder perspective and did not include the full impacts of relocation such as patient out of pocket (OOP) costs or psychosocial costs. OOP costs for relocated people are expected to be over and above the OOP costs experienced by dialysis patients in general. The impacts of relocation are substantial and often manifest in missed treatments and poorer health outcomes [51, 52]. However, these costs resonate well beyond the individual and include the extended family and community.

## Limitations

Retrospective observational hospital data presents challenges for cost analysis. Costs are based on activity and researchers must determine reasons for low or no activity. Where there was a priori knowledge of missing dialysis attendance for DxMoC4 and 5 (nonignorable missing values [53]) we made every effort to gather this information from other sources and link the data sets. We did not interpolate data nor impute costs as we could not be certain activity had occurred. This may under-estimate costs associated with dialysis treatments, although in those DxMoC where dialysis attendance was lower, hospitalisation activity and costs were higher. This supports the assumption that the lower observed activity and costs were not due to missing data. However we accept that this does not necessarily imply cause and effect and that there are other reasons for hospitalisations. For instance admissions for infections, respiratory disorders and metabolic complications also rated highly. In addition, a local study found some remote residing patients voluntarily move to urban areas as their health needs increase [24] and we acknowledge clinician and patient self-selection may limit access of frail and complex patients (who are more likely to be hospitalised) to remote models of care, particularly where there is limited facility capacity and waiting lists exist.

## Conclusion

Aboriginal patients and communities have long advocated for services closer to home, arguing for equitable and accessible services and pointing to literature documenting the psychosocial and economic imposts of relocation [54–58]. A reluctance to establish dialysis services in remote areas can be understood, given the increased resources associated with service delivery logistics and initial infrastructure expenditure. Concerns also exist regarding the quality of care available for remote residing patients [59–61]. In the NT, this reluctance is coupled with the perception that the already high rates of hospitalisations experienced by Aboriginal people on RRT, would be exacerbated further by distance and limited access to tertiary and specialist services in remote areas, resulting in significant increases in health service costs [23, 24].

Our study however, demonstrates that the increased costs associated with remote dialysis service delivery are more than offset by reduced downstream health service use (hospital admissions, ED visits) and costs. Valid economic assessments necessitate a consideration of the broader impacts of treatment access (or lack of), such as requirement for community infrastructure, logistics and cost of relocation, and importantly, the challenges faced by patients.

It is critical that these factors are not ignored in the design of models of care. The shaping of equitable and accessible service delivery models requires community consultation and consumer input as well as an examination of the full cost impacts of different dialysis models.

This study demonstrates the importance of understanding and interpreting the full implications of delivering dialysis services in different locations in order to accurately inform policy decisions. If health systems are to aim for efficient and effective models that meet the needs of the individual, a more holistic approach to the design process is required.

## Supplementary Information

**Additional file 1.**

## Data Availability

The data that supports the findings of this study are available from the Northern Territory Department of Health but restrictions apply to the availability of these data, which were conditions of the Ethics approval and so are not publicly available. Data are however available from the authors upon reasonable request and with permission of the Northern Territory Department of Health.
