# Supplementary material for: Do remote dialysis services really cost more? An economic analysis of hospital and dialysis modality costs associated with dialysis services in urban, rural and remote settings
Source: BMC Health Serv Res. 2021 Jun 17;21:582. doi: 10.1186/s12913-021-06612-z (PMC8212525; doi:10.1186/s12913-021-06612-z)
Supplement: Supplementary file 1 — Additional file 1. [file 12913_2021_6612_MOESM1_ESM.pdf]

Title

Do remote dialysis services really cost more? An economic analysis of hospital and dialysis modality costs associated with dialysis services in urban, rural and remote settings

Journal

Submission to the BMC Health Services Research

Authors:

Gillian Gorham[1], Kirsten Howard[2], Joan Cunningham[1], Federica Barzi[1], Paul Lawton[1], Alan Cass[1]

[1]Menzies School of Health Research, Charles Darwin University, Darwin Australia

[2]Sydney School of Public Health, The University of Sydney, Sydney Australia

Corresponding author:

Gillian Gorham

Menzies School of Heath Research; PO Box 41096; Casuarina 0810; Darwin Australia

ORCID ID: 0000-0002-9814-2204

Email: gillian.gorham@menzies.edu.au;

Phone: +61 8 8946 8529

Electronic Supplementary Material

This supplement includes the Material and Methods from the "Dialysis attendance and health care utilisation associated with urban, rural and remote models of dialysis care" study (under review at time of submission) which preceded this study (***Do remote dialysis services really cost more? An economic analysis of hospital and dialysis modality costs associated with dialysis services in urban, rural and remote settings).*** The base dataset created for the preceding study was also used for this study.

Methods

Australia's health system is a mixture of publicly and privately funded services. Dialysis treatments in the Northern Territory are fully publicly funded under a case mix model based on coded discharge hospital data. All haemodialysis treatments are entered into the NT Department of Health's (DoH) Admitted Patient Care (APC) (hospital) dataset. We conducted a retrospective analysis using linked clinical and administrative data sets to examine health service utilisation (dialysis attendance rates, hospital admissions, days in hospital and emergency department presentations) stratified by service type and location of patients receiving dialysis in the NT between 2008 to 2014.

Study cohort definition

The full database population was derived from the DoH's APC (hospital) dataset combined with the Australia and New Zealand Dialysis Transplant Registry (ANZDATA) dataset.

The APC hospital dataset contains individual episodes of patient care for the five parent hospitals and several satellite services in the NT, from the beginning of consistent electronic record keeping (1991). It includes demographic details of the individual (age, ethnicity, residence) and the hospital (hospital code, ward/s), as well as admission/separation codes and diagnosis and procedure codes (primary and up to 49 secondary codes) based on the International Classification of Diseases version 10, Australian Modification (ICD 10AM). The one private hospital in the NT does not deliver dialysis treatments. ANZDATA is the data repository for people receiving maintenance renal replacement treatment (RRT) in Australia and New Zealand and contains patient level administrative and clinical data, based on an annual census from participating renal units.

The full database population included: 1) any individual from the APC dataset with an ICD 10AM diagnosis or procedure code for dialysis or transplantation (Table S1) between the years 2000 and 2015 (n= 2844); and 2) any individual from the ANZDATA dataset who registered as ever having dialysis in the NT between 2000 and 2015 (n=1390).

Table S1 includes the ICD 10AM diagnostic and procedure codes used to identify RRT admissions and create the 'database population' from the NT Department of Health Admitted Patient Care Hospital Dataset 1991-2015.

Table S1 ICD 10AM codes used to identify database population from hospital dataset

**ICD 10AM Procedure Code**
13100-00=Haemodialysis
13100-01=Intermit Haemofiltration
13100-02=Cont Haemofiltration
13100-03=Intermit Haemodialfiltration
13100-04=Cont Haemodialfiltration
13100-06=Peritoneal Dialysis (PD) short term
13100-07=Intermit PD long term
13100-08=Cont PD long term
13109-00=Insertion and fixation of PD catheter
13109-01=Replacement of indwelling catheter
13110-00=Removal of indwelling catheter
13112-00=PD with temporary catheter
36503-00=Kidney transplant
36503-01=Reimplantation of kidney
90351-00=Removal of temporary PD catheter
**ICD 10AM Diagnosis Code**
T85.71=Infection and inflam reaction due to PD catheter
T86.1=Transplant rejection
Y84.1=Kidney dialysis (Other med proc as a cause)
Z49.0=Preparatory care for dialysis
Z49.1=Extracorporeal dialysis
Z49.2=Other dialysis
Z94.0=Kidney transplant status
Z99.2=Dependence on renal dialysis

The two datasets were linked by a third-party jurisdictional data linkage agency (SA/NT Datalink) following standard ethical systems and protocols. SA/NT Datalink is an independent agency based at the University of South Australia. Using probabilistic matching, de-identified individuals across data sets were linked and assigned a unique identifier. Due to the voluntary nature of the ANZDATA collection, a one to one (1:1) match with the hospital dataset was not expected and one hundred and thirty-two (132) individuals in the hospital dataset were not present in the ANZDATA set. Sixty-seven (67) individuals in ANZDATA dataset did not match any individuals in the hospital data set. These 67 individuals were excluded as hospital activity data was not available for analysis. The datasets included all hospital admission and registry data for eligible patients. The full database population of 2844 individuals were then linked with activity data from two additional data sets: a) interstate patient travel information (n=171); and b) dialysis data from (n=189) individuals receiving care in DxMoC4 and DxMoC5. This was necessary because inconsistencies in data entry for these models led to some gaps in attendance data, however, manual compilation of activity between 2008-2014 was possible and linkage with the hospital data set was undertaken by an independent linker not associated with the project.

Patients were excluded if they were 16 years and younger at 2008 or did not have at least one admission after 2008 – to exclude patients who left the NT and were therefore not eligible for inclusion. All available admission data pre 2008 was also retained for the purposes of identifying home residence and health status (comorbidities) prior to commencing dialysis.

The final study population (n=1003) included individuals who had any RRT for more than three months continuously (to eliminate acute and short-term dialysis support including patients visiting from elsewhere on holidays), between the years 2008 to 2014. This date range was chosen as some models of care only became fully established after 2008 and the additional activity data (for DxMoC4 and DxMoC5) was provided to the end of 2014. Restricting the analysis of patterns of health service utilisation to 2008 to 2014 ensured sufficiently robust activity data across all models was available for the analysis (Figure S1).

Fig. S1 Patient selection flow chart 2008-2014

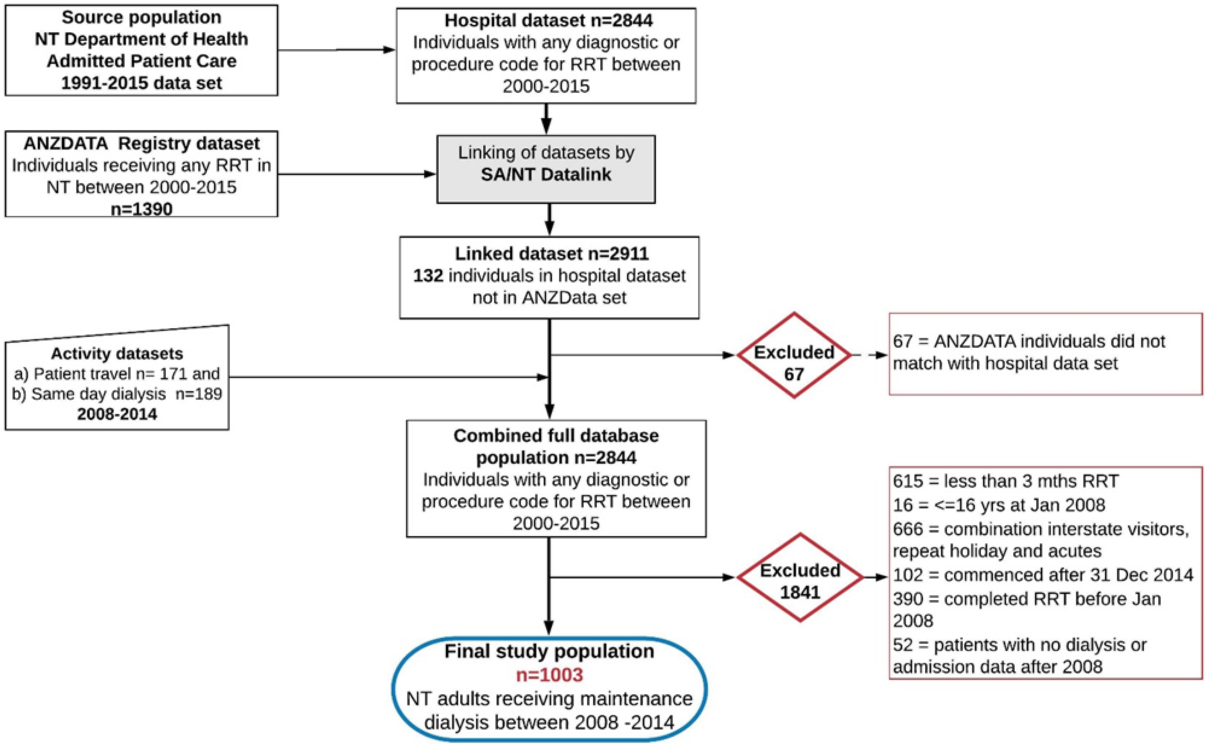

NB: Hospital dataset (n= 2844) equals Combined full database poulation (n=2844) but are not exact patient match

Each admission was aligned with a RRT treatment option of haemodialysis (HD), peritoneal dialysis (PD) or transplant based on diagnosis or procedure codes (Table S2). Relevant diagnosis and procedure codes were present for all patients for at least one admission a year but not all admissions contained a RRT relevant code.

Table S2 ICD 10AM diagnostic and procedure codes used to establish dialysis model of care

**ICD 10AM Procedure Code**
13100-00=Haemodialysis
13100-07=Intermit PD long term
13100-08=Cont PD long term
13109-01=Replacement of indwelling catheter
13110-00=Removal of indwelling catheter
**ICD 10AM diagnosis Code**
Z49.1=Extracorporial dialysis
Z94.0=Kidney transplant status
**AR-DRG Code**
L61Z=HD
L68Z=PD

Table S3 identifies the ICD 10AM codes (collapsed to group level) used to identify and map comorbid conditions for each admission. Once a comorbid condition was present it was carried forward to other admissions if not already present. Comorbidities were chosen for their relevance as kidney disease risk factors and concurrent chronic diseases considered to have a significant impact on health outcomes of kidney patients.

Table S3 ICD 10AM codes used to identify presence of comorbid conditions for each admission in Final Study Population 2008-2014

	**Endocrine, nutritional and metabolic diseases and disorders of circulatory system**	
**Variable**	**ICD 10 Group**	**High level description**
**Diabetes**	E10-E14	Diabetes mellitus
	O24	Diabetes mellitus in pregnancy
**Obesity**	E65-E68	Overweight, obesity and other hyperalimentation
**Hypertension**	I10-I15	Hypertensive diseases
**Cardiac disease**	I20-I25	Ischemic heart diseases
	I26-I28	Pulmonary heart disease and diseases of pulmonary circulation
	I30-I52	Other forms of heart disease
**Cerebrovascular disease**	I60-I69	Cerebrovascular diseases
**Vascular disease**	I70-I79	Diseases of arteries, arterioles and capillaries
	I80-I89	Diseases of veins, lymphatic vessels and lymph nodes, not elsewhere classified
	I95-I99	Other and unspecified disorders of the circulatory system

To determine whether an individual relocated for treatment and whether any treatment was received at or closer to home, we identified home address (to suburb level) for each admission episode that occurred in the 24 months prior to commencement of RRT, taking the earliest admission address as the residence pre RRT start. Less than 1% of patients did not have an admission in the 24 months prior to RRT start. Patients were categorized as ‘Relocated’ when they lived outside the urban areas of Darwin and Alice Springs prior to commencement of RRT.

Remoteness of communities was determined by mapping residence pre RRT start to the Modified Monash Model (MMM) which categorises areas according to remoteness. Activity was separated by region, Top End (TE) and Central Australia (CA), to align with health service responsibility in the NT.

Variable definitions (descriptions and calculations) are shown in Table S4.

The intent of our study was to explore the relationship between dialysis model of care and subsequent health care use. Therefore, the dominant model for each patient was determined for each week, based on the model in which the majority of attendance had occurred in the current and preceding two-week period, rolling forward a week at a time. The process, while eliminating extremely frequent movements between models, still enabled the respite model of 2-3 weeks to be captured in the data.

Table S4 Description of available and created Outcome and Exposure variables in Final Study Population data set

Variable	Variable label	Value	Calculation	Type
year	Year of admission	Continuous (2008-2014)	Year of admission date	**Exposure**
indig	Ethnicity (Aboriginal)	Categorical (0= Not Aboriginal; 1=Aboriginal)	Hospital coding - collapsed to 'Aboriginal or Torres Strait Islander' or 'Not'	**Exposure**
gender	Gender (Male)	Categorical (0= Female; 1=Male)	Hospital coding	**Exposure**
region	Region (TE)	Categorical (0=Central Australia; 1=Top End)	Based on hospital coding	**Exposure**
Orig_district	Residence pre RRT stratified by NT Health Service district	Categorical (1=Darwin; 2= Darwin rural; 3=Tiwi Islands; 4=East Arnhem; 5=Daly West Arnhem; 6=Katherine; 7=Alice Urban; 8=Alice rural; 9=Barkly; 10=Central Desert; 11= Western	Based on hospital coding of Locality codes allocated to NT Health Service district	**Exposure**
Reloc_flag	Relocated	Categorical (0=Not relocated; 1=Relocated)	Flagged if NT Health Service District not equivalent to Darwin or Alice Springs	**Exposure**
Orig_MM7	Remoteness of residence pre-RRT start	Categorical (1=Outer regional; 2=Remote; 3=Very remote; 4=Interstate)	Modified Monash Model classification for areas of remoteness *Outer regional= Urban	**Exposure**
admage	Age at date of admission	Continuous (18-84)	Calculated from date of birth and date of admission	**Exposure**
admage_cat	Age at date of admission category	Categorical (0=<30yrs; 1=30-39yrs; 2=40-49yrs; 3=50-59yrs; 4=60-69yrs; 5=>70yrs)	Age at admission stratified into 6 categories	**Exposure**
RRT_start	Date of RRT commencement	Ordinal dd/mm/yyyy - Interval	Used as interval variable to calculate time on RRT and time in study	**Exposure**
dxtime_cat	Time on dialysis at time of admission category	Categorical (0=<1 yr; 1=>1-2yrs; 2=>2-3yrs; 3=>3-4yrs;	Calculated from RRT start date to date of admission and stratified by 12 month periods for first 5 years	**Exposure**
DxMoC	Dominant dialysis model of care	Categorical (0=DxMoC0; 1=DxMoC1; 2=DxMoC2; 3=DxMoC3; 4=DxMoC4; 5=DxMoC5;	Determined by majority model attendance over 3 week rolling period	**Exposure**
MoCyr_tar	Proportion of year exposed to DxMoC	Continuous (0-1.0)	Proportion of year spent in DxMoC (base unit =weeks) minus iLTFU and time interstate	**Exposure**
MoCdxatt_cat	Category of calculated dialysis attendance by DxMoC/year	Categorical (1=High:144-156; 2=Medium:132-143; 3=Low:<132)	Dialysis attendance stratified into high, medium and low attendance	**Exposure**

Table S4 Description of available and created Outcome and Exposure variables (continued)

Variable	Variable label	Value	Calculation	Type
DM	Diabetes Mellitus	Categorical (0=No; 1=Yes)	Presence of ICD-10AM code; if present always present	**Exposure**
CVD	Cerebrovascular Disease	Categorical (0=No; 1=Yes)	Presence of ICD-10AM code; if present always present	**Exposure**
CAD	Cardiovascular Disease	Categorical (0=No; 1=Yes)	Presence of ICD-10AM code; if present always present	**Exposure**
Hyptn	Hypertension	Categorical (0=No; 1=Yes)	Presence of ICD-10AM code; if present always present	**Exposure**
Obesity	Obesity	Categorical (0=No; 1=Yes)	Presence of ICD-10AM code; if present always present	**Exposure**
VascD	Vascular Disease	Categorical (0=No; 1=Yes)	Presence of ICD-10AM code; if present always present	**Exposure**
iLTFU	Intermittent lost to follow up	Categorical (0=No; 1=Yes)	Flagged for each week if attendance data (dialysis and hospital) is absent => 52 weeks for haemodialysis patients	**Outcome**
ltfu_time	Period of intermittent LTFU time	Continuous (0-2.75)	Calculated from iLTFU, minimum of 52 weeks	**Used in calculation of time at risk**
calcMoC_att	Calculated outpatient dialysis attendance by DxMoC/year	Continuous (0-156)	Number of dialysis treatments by DxMoC, divided by time (weeks) in DxMoC x 52 (weeks) to represent yearly rate	**Outcome**
MoChosp_adm	Rate of hospital admissions by DxMoC/year	Continuous (0-40)	Sum of overnight admissions while exposed to a DxMoC	**Outcome**
yrlyhosp_adm	Annual rate of hospital admissions	Continuous (0-40)	Sum of overnight admissions by year per patient	**Outcome**
MoCEDpres	Rate of ED presentations by DxMoC/year	Continuous (0-50)	Sum of Emergency Department presentations while exposed to a DxMoC	**Outcome**
yrlyED_pres	Annual rate of ED presentations	Continuous (0-50)	Sum of Emergency Department presentations by year per patient	**Outcome**
MoC_los	Number of inpatient days by DxMoC/year	Continuous (0-365)	Total days as inpatient while exposed to a DxMoC	**Outcome**
yrly_los	Number of inpatient days /year	Continuous (0-365)	Total days as inpatient by year per patient	**Outcome**
LTFU	Lost to follow-up	Categorical (0=No; 1=Yes)	Based on 'missing' data to end of observation period with no indication of death	**Censoring**
stdy_status	Status at end of study	Categorical (1=Alive; 2=Dead; 3=LTFU)	Censored at end of study as Alive, Dead or LTFU	**Outcome**
